# Comparing the benefits of chemoradiotherapy and chemotherapy for resectable stage III A/N2 non-small cell lung cancer: a meta-analysis

**DOI:** 10.1186/s12957-018-1313-x

**Published:** 2018-01-16

**Authors:** Yuqiao Chen, Xiong Peng, Yuan Zhou, Kun Xia, Wei Zhuang

**Affiliations:** Department of Thoracic Surgery, Xiangya Hospital of Central South University, 410008 Changsha, Hunan People’s Republic of China

**Keywords:** Non-small cell lung cancer, Neoadjuvant chemoradiotherapy, Induction therapy, Meta-analysis

## Abstract

**Background:**

Induction chemotherapy has been shown to improve survival of patients with stage III A/N2 (T1–3, N2, M0) non-small cell lung cancer (NSCLC), followed by resection, but the benefits of neoadjuvant radiotherapy still remain controversial.

**Methods:**

PubMed, Embase, and Cochrane library databases were searched for relevant randomized controlled trials (RCTs) comparing the outcomes of induction chemoradiotherapy over induction chemotherapy, in patients with resectable stage IIIA/N2 NSCLC. Odds ratios (ORs) with corresponding 95% confidence intervals (95% CIs) were calculated using random- or fixed-effects model, and heterogeneity was assessed using *I*^2^ test. Publication bias was examined by funnel plots analysis.

**Results:**

A total of three RCTs met the inclusion criteria of our meta-analysis. The pooled results demonstrated that, in comparison to induction chemotherapy, induction chemoradiotherapy has a significant benefit in tumor response, mediastinal downstaging, and pathological complete response of mediastinal lymph nodes. In addition, no more peri-intervention mortality was detected in patients from chemoradiotherapy group, and a higher number of patients from this group had R0 resection. However, our results did not show any difference between overall survival and progression-free survival after 2, 4, and 6 years of follow-ups, in patients undergoing radiation therapy vs. induction chemotherapy.

**Conclusion:**

Preoperative chemoradiotherapy, as compared to induction chemotherapy alone, is associated with similar peri-intervention mortality, a greater tumor response, mediastinal nodule downstaging, and rate of R0 resection, but does not improve survival of resectable stage IIIA/N2 NSCLC patients.

## Background

Lung cancer is the leading cause of cancer-related deaths worldwide, and non-small cell lung cancers (NSCLCs) constitute more than 75% of all lung cancer cases [1, 2]. Tumors invading the ipsilateral mediastinal lymph nodes (stage IIIA/N2) account for 50% of the locally advanced NSCLCs cases [3–5]. The metastases of NSCLC to ipsilateral mediastinum (N2) lead to heterogeneous diseases grouped into three categories: occult N2, resectable N2, and non-resectable N2 [6]. According to the international NSCLC guidelines, patients with occult N2 disease, discovered during surgical resection, should continue with the planned resection along with mediastinal lymph node resection. But, for patients with non-resectable N2 disease, concurrent chemoradiotherapy is recommended. However, the optimal treatment strategy for potentially resectable stage IIIA/N2 NSCLC remains controversial, and the prognosis is unsatisfactory [4].

Previous studies have indicated that induction chemotherapy has the ability to make tumors more operable, improve the likelihood of a complete resection, and eradicate the chances of distant micro-metastases, thereby improving the final survival [7–9]. These findings led to the inclusion and recommendation of induction chemotherapy for resectable stage IIIA/N2 patients before their surgery. Since the publication of these recommendations, neoadjuvant chemoradiotherapy has also been developed, thereby establishing a need to test whether chemoradiotherapy is more beneficial than induction chemotherapy alone. Several trials have been undertaken to study the safety and efficacy of induction approaches by combining chemotherapy and radiotherapy [10–15]. Especially, Shah et al. [16], in 2012, reported a meta-analysis comparing neoadjuvant chemoradiotherapy with chemotherapy alone for potentially operable stage IIIA/N2 NSCLC. They showed that neither regimen had a benefit over the other in terms of OS. However, among the seven studies included in their meta-analysis, only two were randomized control trials with quantitative analysis. In fact, the study by Thomas et al. included a substantial proportion of stage III B (T4 N1–3 M0 or T1–4 N3 M0) patients [10], which reduced the power of the results for stage IIIA/N2 patients. In addition, other studies [13, 14] have tried to compare these two regimens with variable results. Thus, we performed this updated meta-analysis by specifically including only randomized control trials of patients exclusively diagnosed with stage IIIA/N2 NSCLC, to ascertain whether addition of preoperative radiotherapy to chemotherapy would improve the survival outcome in these stage IIIA/N2 patients.

## Material and methods

### Study selection

**Fig. 1:**
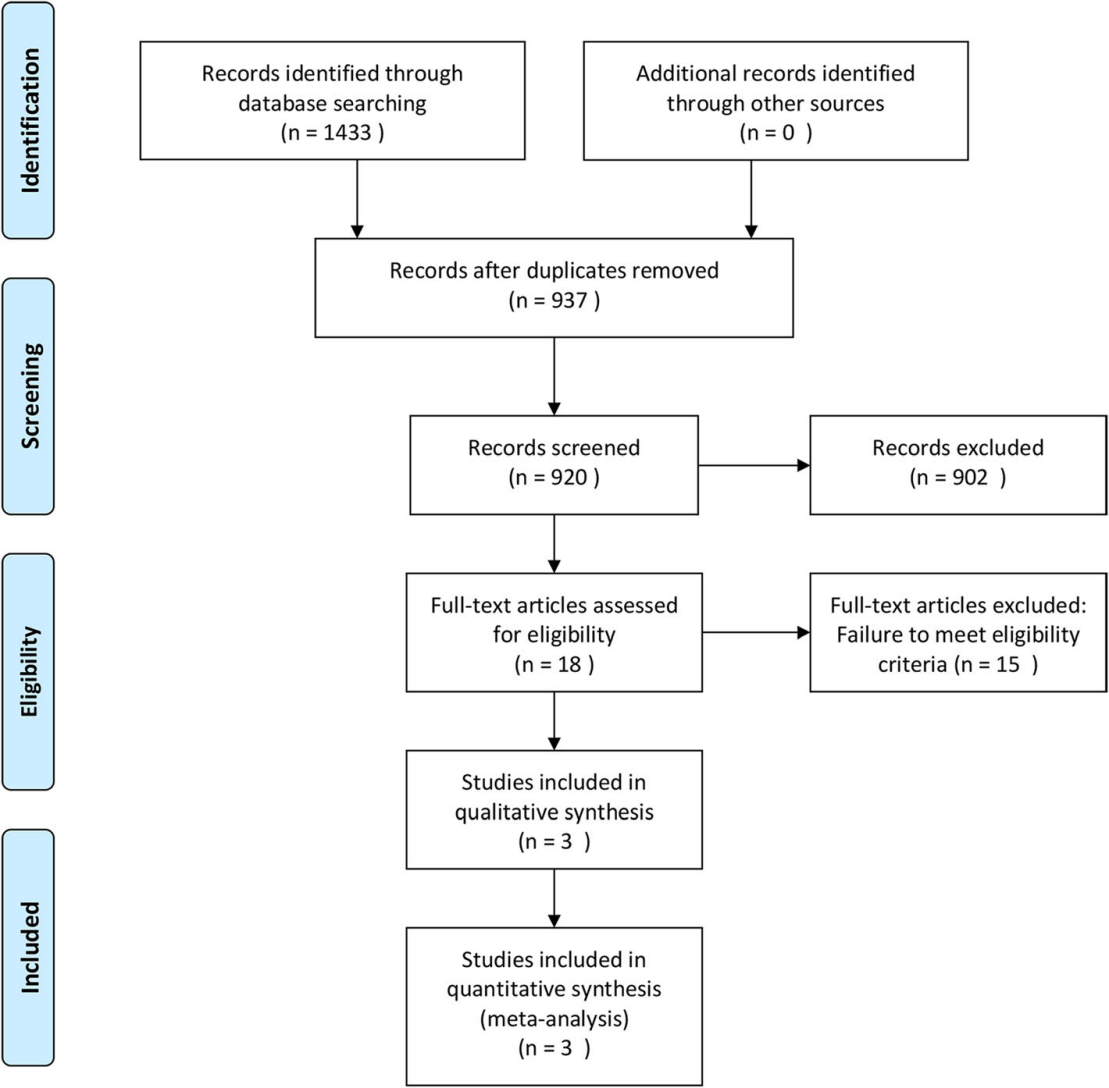
Flow chart depicting the study selection and screening process

Two authors (Chen and Zhou) independently searched PubMed, Embase, and Cochrane Library databases up to July 2017, using the following medical subject heading (MeSH) terms: (1) “non-small cell lung cancer or NSCLC,” (2) “induction therapy or neoadjuvant therapy,” (3) “chemotherapy or chemoradiotherapy,” and (4) “resection or surgery.” The full texts of potentially eligible studies were retrieved and examined to determine which studies met the following inclusion criteria: (a) randomized control trials, (b) studies comparing the use of preoperative induction chemoradiotherapy with chemotherapy alone in the treatment of resectable stage IIIA (T1–3, N2, M0) non-small cell lung cancer patients, and (c) articles published in English, before July 2017. All the relevant clinical studies were manually selected based on titles and summary analyses. Articles reporting studies unrelated to our topic of interest were excluded. Ultimately, only three RCTs fulfilled the eligibility criteria (Fig. 1). All the disagreements about study selection processes were resolved by discussion and consensus with an independent expert (Zhuang).

### Data extraction

Two authors (Peng and Xia) independently extracted the following information from the eligible studies: title, publication year, authors, number of patients, chemotherapy and chemoradiotherapy regimen, and outcomes. The outcome measures included tumor response, pathological complete response, mediastinal nodule downstaging, pathological complete response of mediastinal lymph node, progression-free survival (PFS), and OS at 2, 4, and 6 years. In some cases, the data were extracted from figures, if they were not directly reported in the text or tables. Disagreements about data extraction were also resolved by discussion and consensus with another author (Zhuang).

### Assessment of methodological quality

The quality of the included studies was assessed independently by two authors (Chen and Zhou). Risk of bias among randomized controlled trials (RCTs) was evaluated by RevMan 5.2.10 software (Cochrane Collaboration, UK), using the following domains: sequence generation, allocation sequence concealment, blinding, incomplete outcome data, selective outcomes reporting, and other potential sources of bias. Each domain was classified as low, high, or unclear risk. RCTs were classified into level A (each of the criteria were appropriate), level B (most of the criteria were appropriate), and level C (most of the criteria were not appropriate).

### Data analysis

All the statistical analyses were performed using RevMan v5.2 software (The Cochrane Collaboration, Copenhagen, Denmark). Odds ratios (ORs) with 95% confidence intervals (CIs) were calculated for dichotomized data and a *p* value of ≤ 0.05 represented statistical significance. Heterogeneity in the data was evaluated using *I*^2^ statistics, where an *I*^2^ value of > 50% defined substantial heterogeneity, according to the Cochrane Handbook for Systematic Reviews of Interventions v5.1.0. The fixed-effects model was used for assessing outcome when heterogeneity was low (*I*^2^ < 50%), while random-effects model analysis was performed in cases with substantial heterogeneity (*I*^2^ > 50%). Sensitivity analysis was conducted to confirm the robustness and reliability of the results, and potential publication bias was assessed using funnel plot analysis.

## Results

### Search strategy

Among the 1433 articles identified through the initial search, 18 studies were considered potentially eligible for inclusion. Further full-text analysis led to the exclusion of another 15 studies, and thus, three RCTs [10, 12, 13] were finally included in our meta-analysis (Fig. 1). These three studies included a total of 334 patients, where 157 underwent induction chemotherapy and 177 underwent induction chemoradiotherapy. The characteristics of all three included studies are shown in Table 1.

**Table 1:** Summary of three randomized controlled trials included in the meta-analysis

Study	Publication year	Stage	CT regimen	CRT regimen	Number of patients		Median survival	
					CT	CRT	CT	CRT
Girard et al.	2010	cN2 IIIA	GC	VP or PC + Con RT(46Gy)	14	32	24.2	–
Katakami et al.	2012	pN2 IIIA	DC	DC + Con RT(40Gy)	28	28	29.9	39.6
Pless et al.	2015	pN2 IIIA	DP	DP + Seq RT(44Gy)	115	117	26.2	31.7

Abbreviations: *GC* gemcitabine + cisplatin, *DP* docetaxel + cisplatin, *DC* docetaxel + carboplatin, *VP* vinorelbine + cisplatin, *PC* paclitaxel + carboplatin, *Seq RT* sequential radiochemotherapy, *Con RT* concomitant radiochemotherapy

### Outcome assessments

#### Tumor response and pathological complete response

**Fig. 2:**
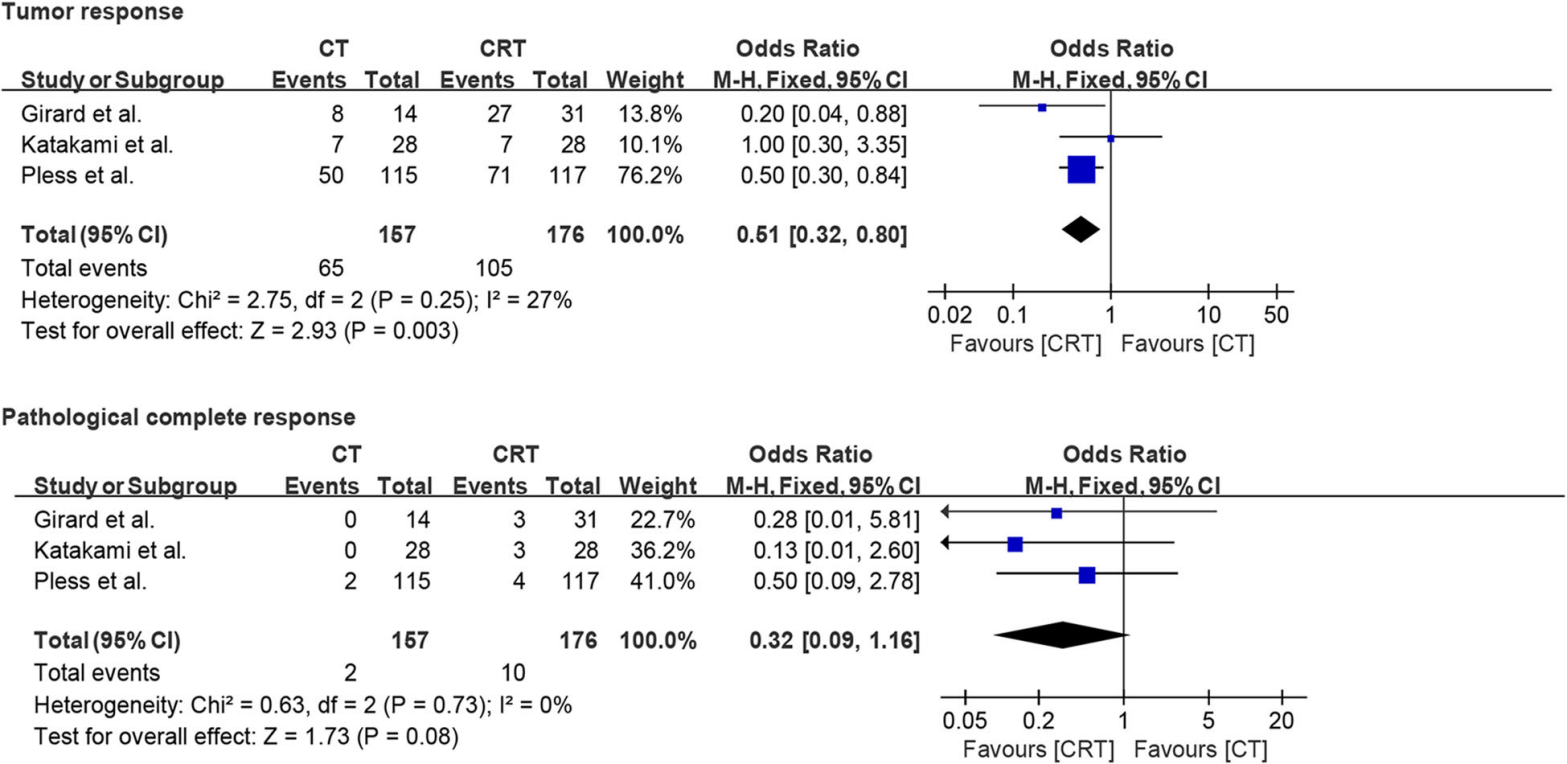
Forest plots comparing tumor response and pathological complete response in patients who received induction chemotherapy or induction chemoradiotherapy

Our meta-analysis, based on all three RCTs reporting data about tumor response and pathological complete response, revealed that induction chemoradiotherapy has a significant benefit in terms of tumor response (OR = 0.51, *p* = 0.003). Similarly, more patients had a pathological complete response after induction chemoradiotherapy than that after induction chemotherapy, but the difference was not statistically significant (OR = 0.32, *p* = 0.08, *I*^2^ = 0%). Importantly, no evidence of significant heterogeneity between the RCTs (*I*^2^ = 27% and *I*^2^ = 0%) was observed (Fig. 2).

#### Mediastinal nodal downstaging and mediastinal lymph nodes pathological complete response

**Fig. 3:**
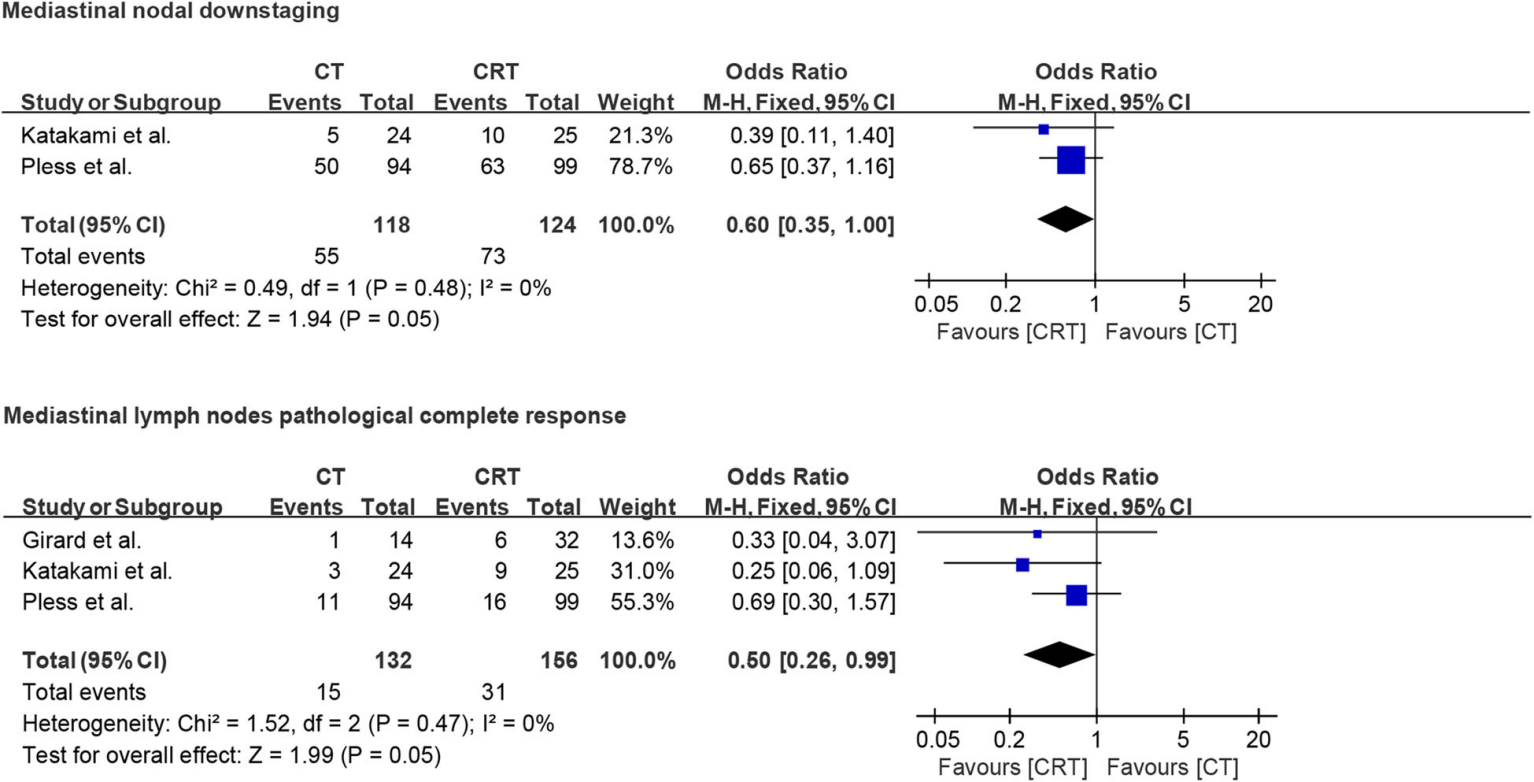
Forest plot comparing nodule downstaging and mediastinal pathological complete response in patients who received induction chemotherapy or induction chemoradiotherapy

Only two studies reported data about mediastinal nodal downstaging from N2 to N1 or N0, while nodal downstaging from N2 to N0, also known as mediastinal lymph node pathological complete response, was reported in all three studies. Interestingly, pooled analysis demonstrated that induction chemoradiation compared to induction chemotherapy had a significant benefit not only in nodule downstaging (OR = 0.60, *p* = 0.05) but also in mediastinal lymph node pathological complete response (OR = 0.50, *p* = 0.05). There was no evidence of significant heterogeneity between the studies (*I*^2^ = 0% and *I*^2^ = 0%; Fig. 3).

#### Incidence rate of patients not receiving surgery after neoadjuvant treatment

**Fig. 4:**
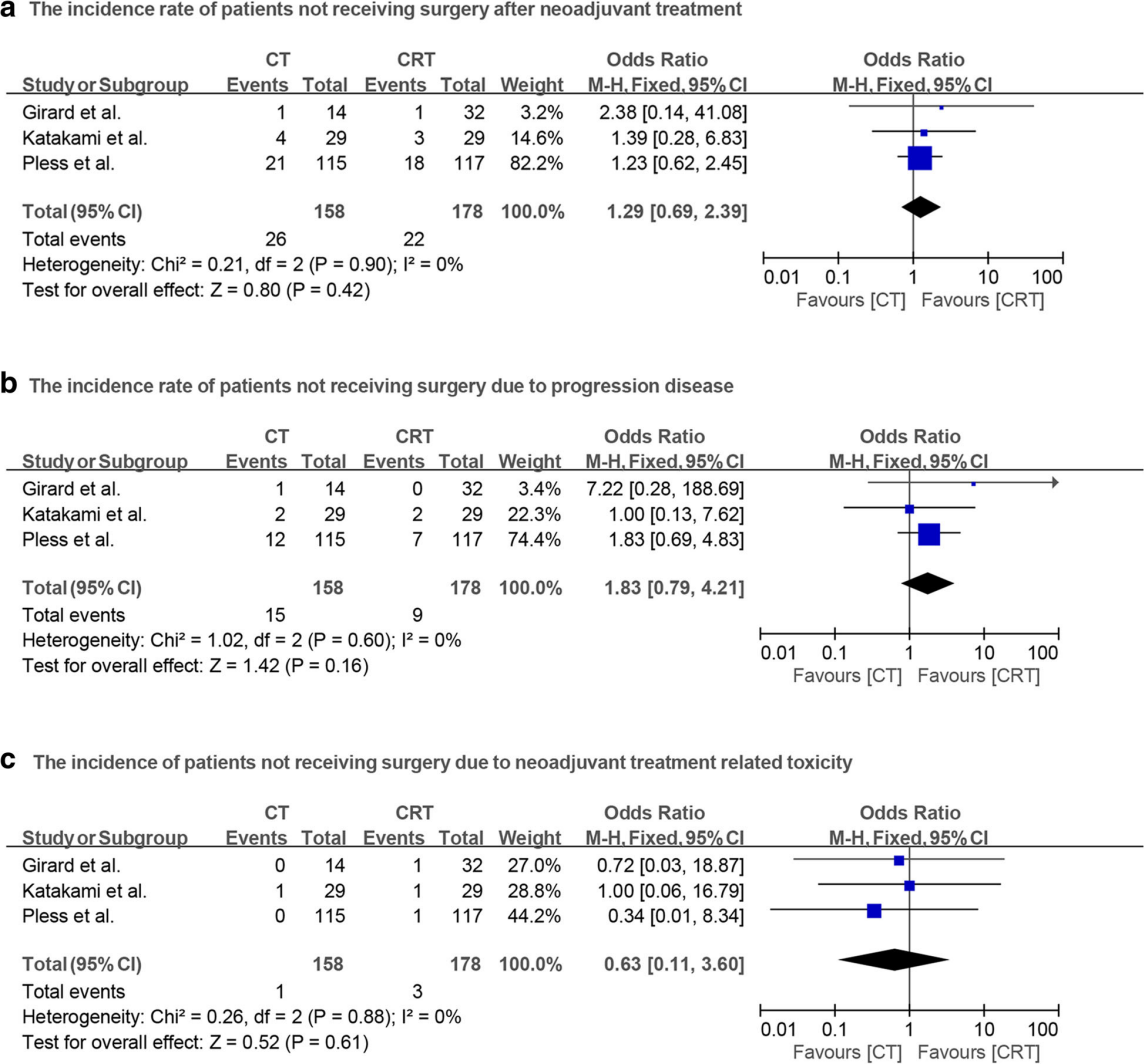
**a** Forest plot comparing the incidence of patients who did not have surgery after neoadjuvant therapy in chemotherapy and chemoradiotherapy groups. **b** Forest plot comparing the incidence of patients not receiving surgery due to progression disease in two groups. **c** Forest plot comparing the incidence of patients not receiving surgery due to neoadjuvant therapy related toxicity in two groups

The study by Pless et al. [14] had 18.3% (21/115) patients in the chemotherapy group and 15.4% (18/117) patients in the chemoradiotherapy group, who did not receive surgery. Similarly, the study by Katakami et al. [13] had 13.8% (4/29) and 10.3% (3/29) patients in the chemotherapy and chemoradiotherapy groups, respectively, who did not receive surgery. Also the study by Girard et al. [11] showed 7.1% (1/14) and 3.1% (1/32) patients in the chemotherapy and chemoradiotherapy groups, respectively, who did not receive surgery. The pooled results from all three RCTs demonstrated that there is no statistically significant difference between the incidence rate of patients not undergoing surgery between both groups, along with no significant heterogeneity (OR = 1.29, *p* = 0.42; *I*^2^ = 0%; Fig. 4a). Progression disease and toxicity of neoadjuvant therapy were the most common reasons for patient excluded from surgery after induction therapy. Again, no significant difference was observed between the patients in both groups, who were excluded from surgery due to progression disease and toxicity of neoadjuvant therapy (OR = 1.83, *p* = 0.16; *I*^2^ = 0%; Fig. 4b; OR = 0.63, *p* = 0.61; *I*^2^ = 0%; Fig. 4c).

#### R0 resection

**Fig. 5:**
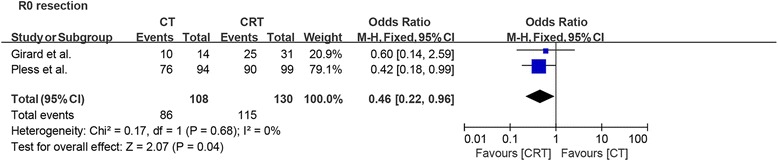
Forest plot comparing R0 resection in patients who received induction chemotherapy or induction chemoradiotherapy

The information about R0 resection was described only in two studies, and our meta-analysis revealed that more patients had an R0 resection after chemoradiotherapy than patients after chemotherapy (OR = 0.46, *p* = 0.04). We did not find any evidence of significant heterogeneity between these two studies (*I*^2^ = 0%; Fig. 5).

#### Overall survival and progress-free survival

**Fig. 6:**
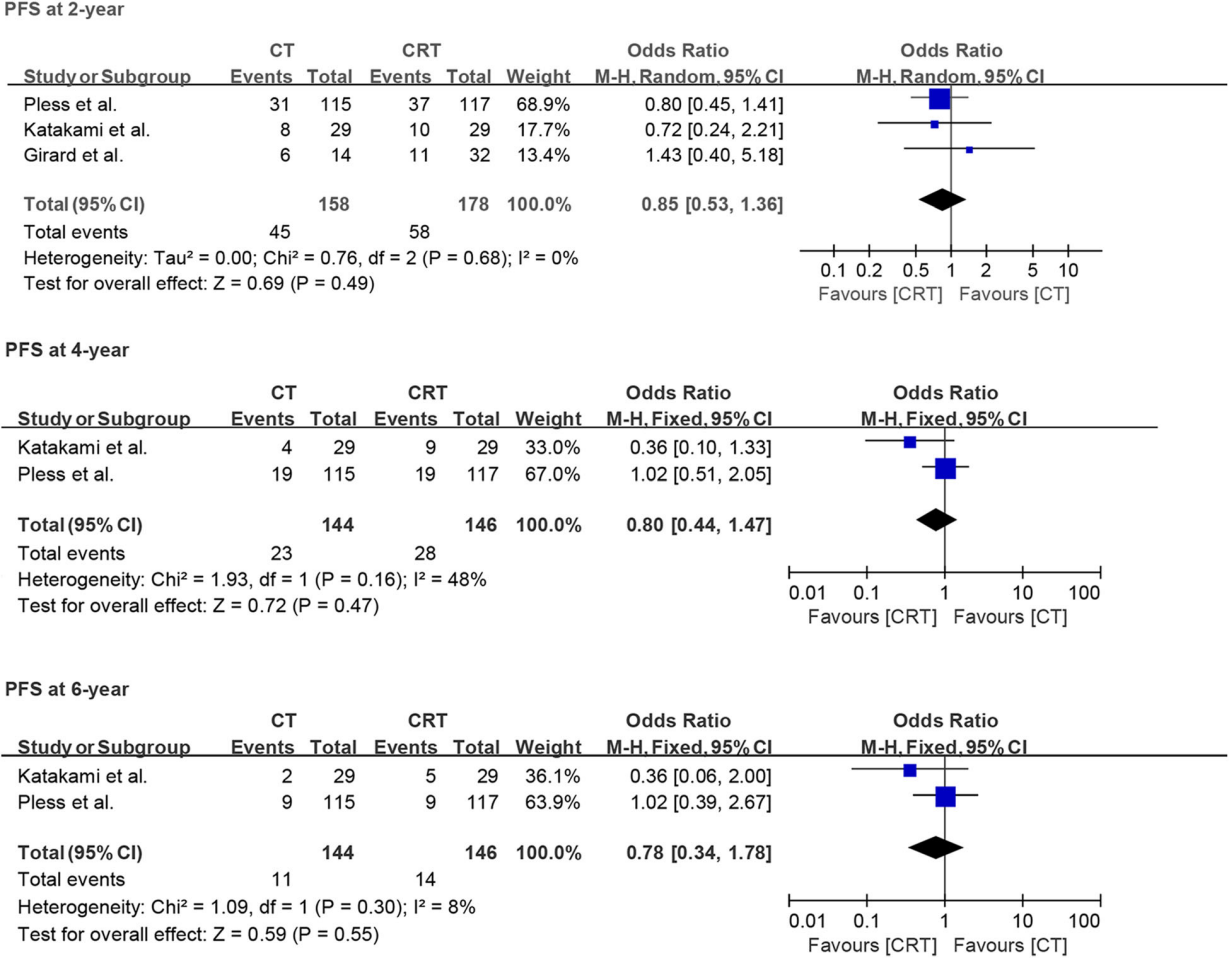
Forest plot comparing progression-free survival at 2, 4, and 6 years in patients who received induction chemotherapy or induction chemoradiotherapy

**Fig. 7:**
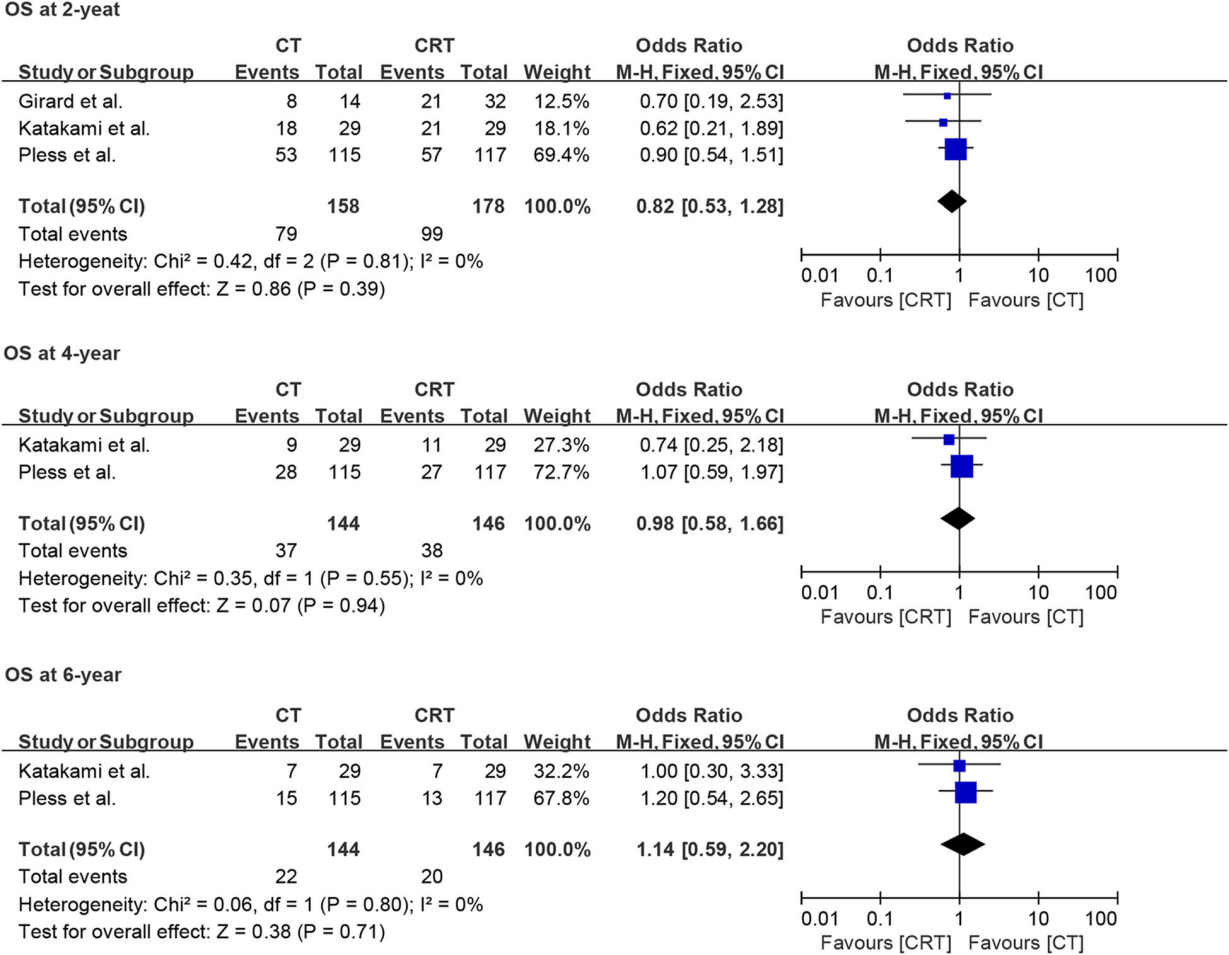
Forest plot comparing overall survival at 2, 4, and 6 years in patients who received induction chemotherapy or induction chemoradiotherapy

All studies reported information about 2-year OS and PFS, while only two studies reported 4- and 6-year OS and PFS. Based on fixed-effects model analysis, we observed no statistically significant benefit of induction chemoradiotherapy over induction chemotherapy for PFS at 2, 4, and 6 years of follow-up (OR = 0.85, *p* = 0.49, *I*^2^ = 0%; OR = 0.8, *p* = 0.47, *I*^2^ = 48%; OR = 0.78, *p* = 0.55, *I*^2^ = 8%; Fig. 6). A similar pattern of no benefit was observed for OS at 2, 4, and 6 years of follow-up (OR = 0.82, *p* = 0.39, *I*^2^ = 0%; OR = 0.98, *p* = 0.94, *I*^2^ = 0%; OR = 1.14, *p* = 0.71, *I*^2^ = 0%; Fig. 7).

### Analysis of methodological quality, publication bias, and sensitivity

**Fig. 8:**
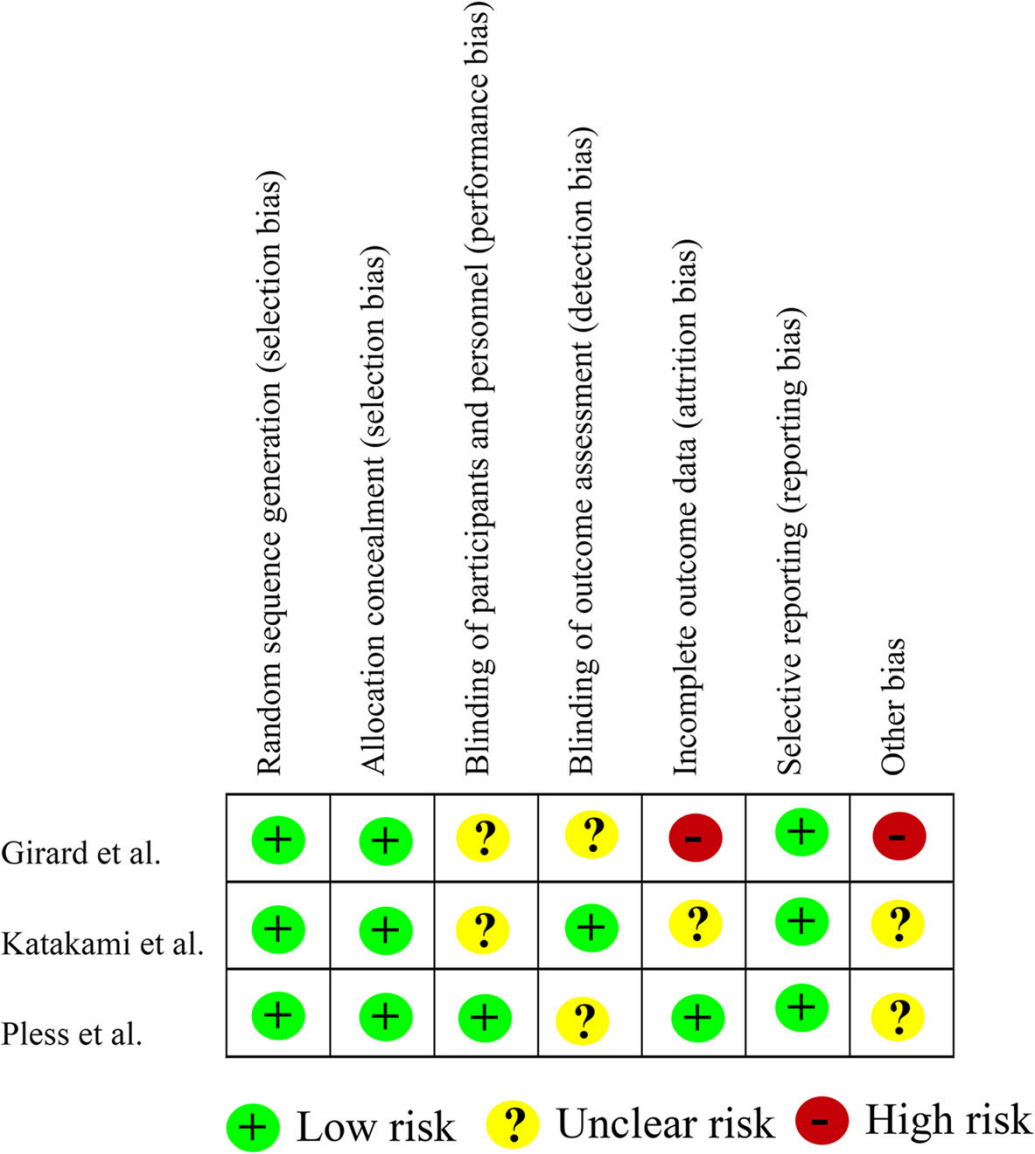
Risk of bias analysis of the included randomized controlled trials

**Fig. 9:**
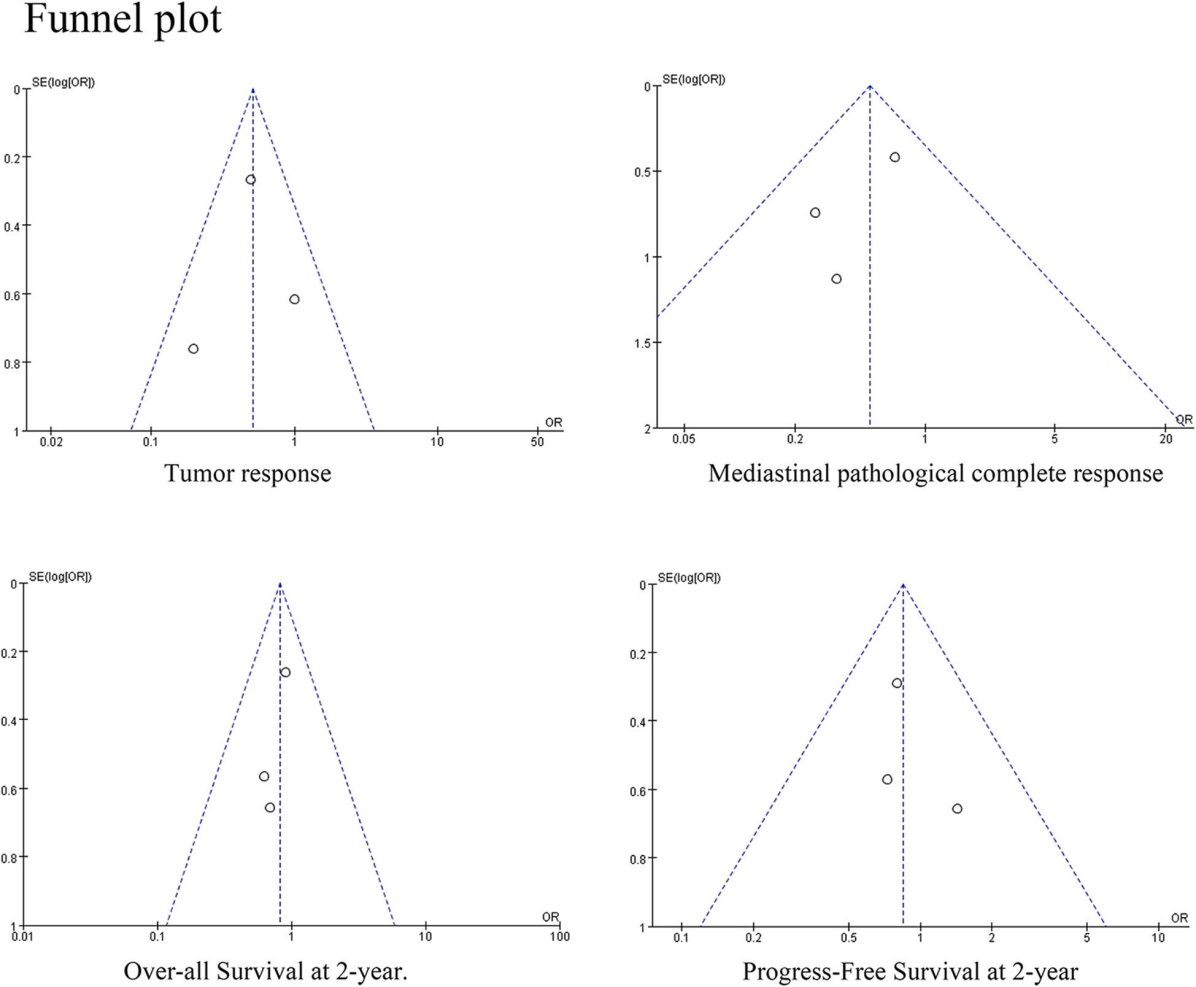
Funnel plot analysis to assess publication bias

The methodological quality of included RCTs was assessed, as shown in Fig. 8. Specifically, the quality of the RCT by Girard et al. was of level B, due to incomplete outcome data that resulted in high attrition bias. However, the other two RCTs were assessed as level A. Next, funnel plots analysis indicated no evidence of publication bias, as shown in Fig. 9. The sensitivity analyses of our data with fixed- and random-effects models showed robustness and reliability (data not shown).

## Discussion

Previous studies have indicated that patients with resectable N2 disease have poor outcomes after resection alone [17, 18]. These studies suggest that multimodal therapy, including systemic treatment for distant control (chemotherapy), is essential in these patients. Reports also indicate that preoperative chemotherapy might make tumors more operable, improve the likelihood of complete resection, eradicate the chances of distant micro-metastases, and finally improve OS [7–9]. However, with the advent of radiotherapy, another local treatment, the question arises of whether its addition to chemotherapy can have any additional benefits for patients with stage IIIA/N2 NSCLC. Currently, the answer seems debatable.

Importantly, based on the data from our meta-analysis, neoadjuvant chemoradiotherapy had an advantage for patients with stage IIIA/N2 NSCLC, in terms of tumor response, mediastinal downstaging, and a pathological complete response in mediastinal lymph nodes. All these benefits indicated local efficacy of radiotherapy. Interestingly, more patients had R0 resection after chemoradiotherapy than after chemotherapy alone. Moreover, a low heterogeneity among the included three RCTs reinforced the robustness of our result. In addition, all patients in this meta-analysis, except 13/46 patients from the trial by Girard et al. [11], had pathological proof of N2-involvement, which provided additional credibility to our results. Although, previous studies have shown that mediastinal downstaging and increased rate of complete resection followed by neoadjuvant therapy are prognostic factors for better survival in patients with stage IIIA/N2 NSCLC [19–21], our pooled results demonstrated that addition of radiotherapy into chemotherapy was not superior to neoadjuvant chemotherapy alone, in terms of PFS and 2-, 4-, and 6-year OS. It is important to mention here that pooled results from our study were similar with those from the large retrospective study by Yang et al. [15], which included 1362 patients with clinical IIIA (T1–3N2M0) disease. Their results showed that downstaging from N2 to N0/N1 was more common with induction chemoradiation than with induction chemotherapy (58 vs 46%), but the 5-year survival of patients was similar between both groups (41 vs 41%). It has been observed that induction radiation could cause fibrosis and extensive adhesion and may lead to more difficult mediastinal lymph node dissections [22, 23]. The study by Yang et al. revealed that the number of lymph nodes harvested was reduced after adding radiation to induction chemotherapy [15]. The overall examination of fewer lymph nodes may underestimate the post neoadjuvant therapy pathological stage (ypTNM) and can lead to higher incidence of mediastinal downstaging and R0 resection. All these observations may explain why the increased rate of mediastinal downstaging and complete resection did not result in better survival in the chemoradiotherapy group as we had observed in this meta-analysis.

Additionally, not all the patients having induction therapy are eligible for surgery and the main reason for patient exclusion from definitive surgery is progression disease and severe therapy-related toxicity. The pooling results showed no significant difference in patients excluded from definitive surgery between both groups. More specifically, no difference between chemotherapy group and chemoradiotherapy group was observed in terms of progression disease and severe therapy-related toxicity after induction therapy. High incidence of perioperative mortality, ranging from 4 to 9%, after induction therapy has been noted [23–25]. However, perioperative mortality in our study was lower in comparison to that in previous studies. Interestingly, the studies of Girard et al. [11] and Katakami et al. [13] showed no peri-interventional mortality in both chemotherapy and chemoradiotherapy groups. The study by Pless et al. [14] showed mortality rate of 3% (3/94) in patients from the chemotherapy group and 0% in the chemoradiotherapy group. The possible reason for this difference in mortality rate could be that our included studies were published earlier (2000–2007) than other studies like the trials by Thomas et al. [10] and Albain et al. [25] (1994–2003). The changed practice patterns over time, including more strict selection criteria for pneumonectomy, strategies to protect the bronchial stump, perioperative fluid administration, and use of three-dimensional radiation planning, can explain the better outcomes reported in the present meta-analysis [10, 11, 13, 14, 24]. Moreover, previous study by Thomas et al. [10] demonstrated increased surgical mortality after addition of preoperative radiotherapy to induction chemotherapy (9.2 vs 4.5%). But our meta-analysis showed no significant difference between those two groups in terms of postoperative mortality [11, 13, 14].

In summary, addition of radiotherapy into induction therapy did not increase peri-interventional mortality. But it should not be recommended, in addition to chemotherapy before surgery, in patients with stage IIIA/N2 NSCLC, due to high medical expenses and little benefit in terms of survival. It is important to mention that the numerous limitations of the RCTs included in our meta-analysis can hinder the conclusiveness of our data. As our meta-analysis only included three RCTs, our study is susceptible to being underpowered. Indeed, the sample size in two of these RCTs was too small to detect meaningful differences in the outcomes.

## Conclusion

Preoperative chemoradiotherapy, as compared to chemotherapy alone, can increase the pathological response and mediastinal downstaging in patients with resectable stage IIIA/N2 NSCLC, without increasing peri-interventional mortality. However, it does not improve long-term survival. Going forward, additional high-quality randomized controlled trials should be undertaken to further confirm the validity of our results.
